# Elevation of YKL-40 in the CSF of Anti-NMDAR Encephalitis Patients Is Associated With Poor Prognosis

**DOI:** 10.3389/fneur.2018.00727

**Published:** 2018-10-12

**Authors:** Jinyu Chen, Yuewen Ding, Dong Zheng, Zhanhang Wang, Suyue Pan, Teng Ji, Hai-Ying Shen, Honghao Wang

**Affiliations:** ^1^Department of Neurology, Nanfang Hospital, Southern Medical University, Guangzhou, China; ^2^Department of Neurology, The Affiliated Brain Hospital of Guangzhou Medical University, Guangzhou, China; ^3^Department of Neurology, 39 Brain Hospital, Guangzhou, China; ^4^Department of Neurology, Randall Children's Hospital, Legacy Health, Portland, OR, United States; ^5^RS Dow Neurobiology Laboratories, Legacy Research Institute, Legacy Health, Portland, OR, United States

**Keywords:** anti-NMDAR encephalitis, cerebrospinal fluid, YKL-40, modified rankin scale, cytokines

## Abstract

**Objective:** Anti-N-methyl-D-aspartate receptor (NMDAR) encephalitis predominantly affects children and young women; the disease can have a multistage presentation and exhibit a wide variety of neuropsychiatric features. This study aimed to investigate the profile of YKL-40 (Chitinase 3-like 1) in anti-NMDAR encephalitis patients and evaluate its association with modified Rankin Scale (mRS) scores and expression of inflammatory cytokines.

**Methods:** A total of 66 patients were enrolled in this study, 33 with anti-NMDAR encephalitis, 13 with viral meningitis and 20 with non-inflammatory neurological disease. Patients were evaluated to determine mRS scores at disease onset and at the 3 month follow-up; cerebrospinal fluid (CSF) samples were collected in the meantime. CSF levels of YKL-40 and cytokines (TNF-α, IL-6, IL-10) were measured by enzyme-linked immunosorbent assay.

**Results:** CSF levels of YKL-40 and inflammatory cytokines (TNF-α, IL-6, IL-10) were all more highly elevated in patients with anti-NMDAR encephalitis at the acute stage of disease compared with the controls. Levels of CSF YKL-40 were correlated with levels of IL-6 both at disease onset and at the 3 month follow-up. Changes in YKL-40 levels were significantly correlated with improved mRS scores in patients with anti-NMDAR encephalitis.

**Conclusion:** Our study suggests that CSF levels of YKL-40 in patients with anti-NMDAR encephalitis were increased and correlated with clinical mRS scores. This may be reflective of the underlying neuroinflammatory process. YKL-40 demonstrates potential as a possible biomarker for the prognosis of anti-NMDAR encephalitis.

## Introduction

Anti-N-methyl-D-aspartate receptor (NMDAR) encephalitis is a newly recognized acute form of encephalitis caused by anti-neuronal autoantibody; anti-NMDAR encephalitis is potentially lethal and mainly affects children and young women (1, 2). Anti-NMDAR encephalitis can develop with multistage presentation and a wide spectrum of neuropsychiatric features. The early-phase involves nonspecific prodromes such as fatigue, fever and headache. In late-phase, impaired consciousness, cognitive deficits, seizures, profound psychiatric symptoms, dysautonomia, and central hypoventilation often emerge, necessitating intensive care treatment (3–5). One of the challenges for management of anti-NMDAR encephalitis is the lack of available biomarkers for monitoring and predicting prognosis. The hallmarks of neuroinflammation in anti-NMDAR encephalitis should be thoroughly investigated to improve treatment and patient outcomes.

Anti-NMDAR encephalitis is caused by autoantibodies which primarily target the NR1 subunit of the NMDAR. Transformation of B and T lymphocytes is thought to be involved in the inflammatory injury that occurs with anti-NMDAR encephalitis (6). Gliosis, microglia proliferation and IgG deposition are suggested to be the most prominent histopathological features of the disease (7). YKL-40 (chitinase 3-like 1 or HCgp39) is a secreted glycoprotein belonging to the glycosyl hydrolase 18 family. In the central nervous system (CNS), YKL-40 is mostly expressed by microglia, especially when responding to acute and chronic inflammation (8, 9). An increase of YKL-40 in the brain and/or cerebrospinal fluid (CSF) is associated with a variety of immune and inflammatory diseases, particularly neurological and neurodegenerative diseases with an inflammatory component, such as multiple sclerosis (MS), Alzheimer's disease (AD), and stroke (10–12). However, little is known about what inflammatory mediators regulate YKL-40 or the underlying molecular mechanisms behind this regulation. In the present study, we aimed to investigate the YKL-40 profile in patients with anti-NMDAR encephalitis and to evaluate possible associations between YKL-40 and modified Rankin Scale (mRS) scores, as well as the expression of inflammatory cytokines.

## Materials and method

### Patients and controls

This study enrolled 33 patients with anti-NMDAR encephalitis, 13 patients with viral meningitis (VM) and 20 controls. The diagnosis of anti-NMDAR encephalitis was confirmed using criteria which included the presence of clinical manifestations and detection of anti-NMDAR antibodies in the CSF using a cell-based assay (2, 13). Patients with non-inflammatory neurological disease were used as controls, including 15 cases of mild white matter degeneration and 6 cases of movement disorders. The etiologies of viral meningoencephalitis included 7 cases of herpesvirus and 6 cases of varicella zoster virus (Table 1). Within 3 days of admission, all patients were subjected to a lumbar puncture procedure for CSF analysis, patients with anti-NMDAR encephalitis received a follow-up lumbar puncture for CSF re-evaluation 3 months after discharge. All patients with anti-NMDAR encephalitis received tumor screening at least once during hospitalization using computed tomography, magnetic resonance imaging or B-scan. The neurological status of patients was assessed using the mRS (14) at both onset and at the 3 month follow-up after discharge. Treatments for anti-NMDAR encephalitis included first-line immunotherapy, second-line immunotherapy and tumor removal.

**Table 1:** The demographic data of patients with anti-NMDAR encephalitis (*n* = 33), viral meningitis (*n* = 13) and controls (*n* = 20).

**Characteristic**	**Anti-NMDAR encephalitis**	**Viral meningitis**	**Controls**
Gender (female/male)	19/14	5/8	13/8
Age (years, mean ± SD)	34.8 ± 17.5	35.7 ± 15.7	34.81 ± 13.32
Symptom onset (n, %)			
Prodromal symptoms	16 (48.5)	0	–
Psychiatric symptoms	32 (97.0)	2	–
Memory deficits	6 (18.2)	3	–
Speech disturbances	2 (6.0)	0	–
Seizures	22 (66.7)	1	–
Movement disorders	7 (21.2)	0	–
Loss of consciousness	21 (63.6)	0	–
Central hypoventilation	7 (21.2)	0	–
Ovarian teratoma	2 (6.1)	0	–
mRS	4.2 ± 0.9	–	–
3 month mRS	2.7 ± 1.2	–	–
CSF anti-NMDAR antibody	33	0	0
Treatment (n, %)			
First line treatment	22 (66.7)	–	–
Second-line treatment	11 (33.3)	–	–
Onset
YKL-40 (pg/ml)^*^	128.3 (97.0–171.4)	94.7 (77.1–113.6)	82.4 (67.4–93.4)
TNF-α (pg/ml)	6.0 (4.3–11.3)	5.27 (3.4–11.3)	1.1 (0.2–2.2)
IL-6 (pg/ml)	7.3 (3.9–13.4)	4.6 (3.4–6.7)	3.1 (2.7–4.1)
IL−10 (pg/ml	4.9 (3.6–5.8)	3.21 (2.0–5.7)	0.9 (0–1.1)
3 month follow-up
YKL-40 (pg/ml)	121.1 (106.4–173.0)	–	–
TNF–α (pg/ml)	3.5 (2.31–8.3)	–	–
IL-6 (pg/ml)	7.3 (5.7–8.2)	–	–
IL-10 (pg/ml)	2.7 (1.3–4.8)	–	–

* *Data were presented as the median (interquartile range)*.

### Enzyme-linked immunosorbent assay (ELISA)

CSF samples were centrifuged immediately after collection to isolate cells and larger particles, then stored at −80°C for future testing. Commercially available sandwich ELISA kits were used to detect CSF concentrations of YKL-40 (Quantikine ELISA, R&D Systems), TNF-α (Cusabio, Wuhan, China), IL-6, and IL-10 (Bender MedSystems GmbH, Vienna, Austria). ELISA assays were performed according to the manufacturers' instructions for each kit.

### Follow-up evaluation

A total of 15 patients with anti-NMDAR encephalitis received a follow-up evaluation 3 months after discharge. The follow-up evaluation included mRS assessment and CSF tests.

### Statistical analysis

All statistical analyses were performed using SPSS 20.0 (IBM Corp, Armonk, NY, USA). Because the data were non-parametrically distributed, statistical analysis to compare levels of YKL-40 and cytokines between anti-NMDAR encephalitis and control groups was conducted with the Kruskal-Wallis *H*-test. Spearman test was performed to evaluate correlations between CSF cytokines and mRS scores. A receiver Operator Characteristic (ROC) curve was used to evaluate the diagnostic value of CSF YKL-40 between anti-NMDAR encephalitis and controls. A value of *p* < 0.05 was considered statistically significant.

## Results

### Increased CSF levels of YKL-40 and cytokines in patients with anti-NMDAR encephalitis

**Figure 1:**
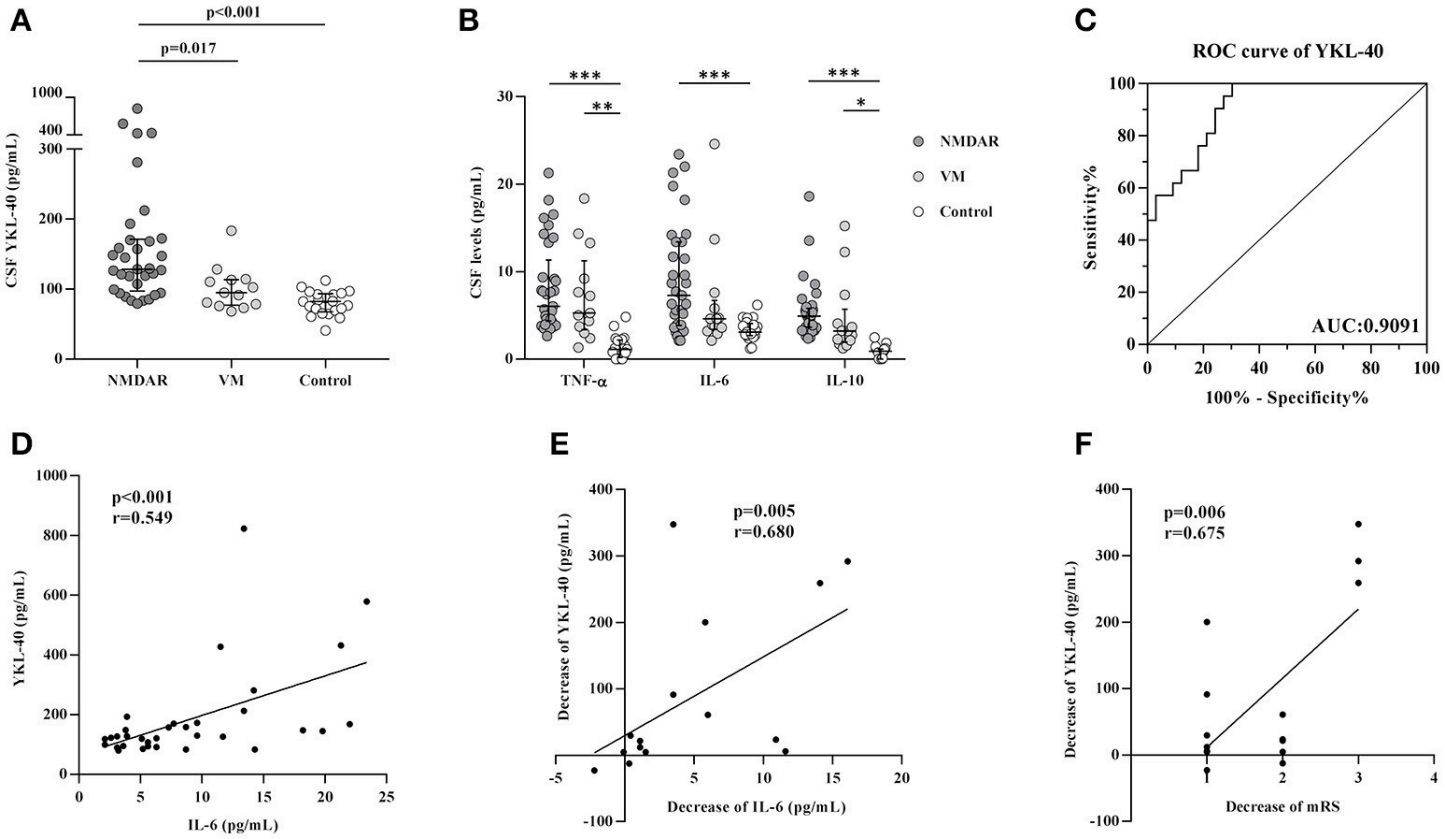
Levels of YKL-40 and cytokines in cerebrospinal fluid (CSF) and association with modified Rankin Scale (mRS). YKL-40 **(A)** and cytokines **(B)** levels in the CSF of patients with anti-NMDAR encephalitis, viral meningitis (VM) and controls; **(C)** Receiver operating characteristic curves for YKL-40 to discriminate anti-NMDAR encephalitis patients from control patients. **(D)** Correlation between YKL-40 levels and IL-6 in anti-NMDAR encephalitis subjects. **(E)** Correlation between decrease of YKL-40 levels and decrease of IL-6 levels in anti-NMDAR encephalitis patients in 3 month follow-up. **(F)** Correlation between decrease of YKL-40 levels and decrease of mRS scores in anti-NMDAR encephalitis patients in 3 month follow-up. *p < 0.05; **p < 0.01; ***p < 0.001.

To investigate the role of YKL-40 in anti-NMDAR encephalitis, we measured the CSF level of YKL-40 in patients with anti-NMDAR encephalitis (*n* = 33), VM (*n* = 13), and controls (*n* = 20) using an ELISA assay. As shown in Figure 1A, the levels of YKL-40 in the CSF of anti-NMDAR encephalitis patients at the onset of disease (median 128.3 pg/mL; interquartile range 97–171.4 pg/mL) were significantly higher than those of VM patients (94.7 pg/mL; 77.1–113.6 pg/mL) and controls (82.4 pg/mL; 67.35–93.35 pg/mL) (*p* = 0.017, *p* < 0.001, respectively).

CSF levels of the pro-inflammatory cytokines TNF-α and IL-6 were significantly higher in anti-NMDAR encephalitis patients than in controls (*p* < 0.001 for both cytokines). The anti-inflammatory cytokine IL-10 was also higher in the anti-NMDAR patients than in controls (*p* < 0.001) (Table 1, Figure 1B). In the acute stage, a positive association was observed between YKL-40 and IL-6 levels (*r* = 0.549, *p* < 0.001) (Figure 1D). ROC curves for YKL-40 were calculated using data from all 66 patients. The area under the ROC curve (AUC) was 0.9091 and the optimal cut-off value was 118.8 pg/mL (Figure 1C), indicate that YKL-40 has a potential diagnostic value of anti-NMDAR encephalitis.

### Altered CSF levels of YKL-40 and cytokines in the follow-up for anti-NMDAR encephalitis patients

CSF levels of YKL-40 in patients decreased significantly from 128.3 pg/mL (97–171.4 pg/mL) (onset) to 121.1 pg/mL (106.4–173 pg/mL) (3 month follow-up) (*p* = 0.015), although the latter was still higher than controls (*p* < 0.001). CSF levels of IL-10, TNF-α and IL-6 at the 3 month follow-up were significantly lower than those at the onset of disease. (*p* < 0.01 for all cytokines). In addition, the decrease in CSF YKL-40 levels was positively associated with the change in IL-6 levels and improvement of mRS scores (Figures 1E,F).

## Discussion

Neuroinflammation is a common feature of anti-NMDAR encephalitis pathology; increasing evidence suggests that inflammatory cytokines and immune cells are important effectors and regulators for the inflammation and autoimmunity associated with this disease (7, 15–18). Autopsy studies using patient brain tissues showed that B cells interact with T cells and macrophages, inducing neuroinflammation (15, 19). YKL-40 levels have been reported in patients with several neuroinflammatory and neurodegenerative diseases (20). It can be expressed in macrophages/microglial cells and has been proposed as a potential marker for ongoing inflammation in a variety of human diseases (21–24). In the present study, we showed that CSF YKL-40 levels were significantly increased in patients with anti-NMDAR encephalitis and the change in YKL-40 levels at follow-up correlated positively with the improvement of mRS. Our results were similar to those reported for YKL-40 from MS and AD studies (25, 26). Elevated YKL-40 levels during acute attacks of anti-NMDAR encephalitis were significantly reduced after effective treatment, which was correlated with the severity of disease (mRS). Reduced levels of YKL-40 in CSF may be attributed to (i) reduced production of YKL-40 from relieved inflammation and tissue damage in the CNS after treatment; and/or (ii) reduced pro-inflammatory effect of YKL-40, which is similar to the possible associated mechanisms of YKL-40 after immunotherapy in MS (27). In our study, the 15 patients that were seen at follow-up were all treated with immunotherapy, which could have affected YKL-40 levels.

We also demonstrated that CSF levels of IL-6, a pro-inflammatory cytokine, significantly increased in anti-NMDAR encephalitis patients. IL-6 is a cytokine that is able to cause a robust, cytokine-driven expression of YKL-40 in astrocytes, which requires use of both the STAT3 and NF-κB binding elements on the YKL-40 promoter (28). Studies have shown that increased IL-6 in CSF and brain tissue could facilitate production of intrathecal anti-NMDAR autoantibody in encephalitis (29, 30). The role of IL-6 in the regulation of YKL-40 is also supported by *in vitro* experiments and observations in patient studies (31). In this study, we found that the changes in IL-6 correlated with changes in YKL-40, suggesting that IL-6 may contribute to YKL-40 expression and the autoantibody-induced neurological deficits observed in anti-NMDAR encephalitis.

Our study has several limitations. First, the sample size was relatively small, caution should be exercised when interpreting these results. In addition, YKL-40 is not disease specific and may be involved in other medical conditions including vascular risk factors and acute infectious diseases, all of which may affect the diagnose value of YKL-40. Finally, this was just a preliminary study, the mechanism of the increases YKL-40 in anti-NMDAR encephalitis patients remain unclear, which should be elucidated with further studies.

## Conclusions

We report, for the first time, the role of YKL-40 in anti-NMDAR encephalitis. Changes in CSF YKL-40 levels positively correlated with improvement of mRS, suggesting that YKL-40 may be implicated in the pathogenesis of anti-NMDAR encephalitis. To fully understand the role YKL-40 is playing in anti-NMDAR encephalitis, additional mechanistic and experimental studies should be conducted in the future.

## Ethics statement

The study was conducted with the approval of the Ethics Committee of Nanfang Hospital, Southern Medical University.

## Availability of data and material

We declared that materials described in the manuscript, including all relevant raw data, will be freely available to any scientist wishing to use them for non-commercial purposes, without breaching participant confidentiality.

## Author contributions

HW, H-YS, and SP co-conceived this study and designed the experiments. JC, DZ, and ZW collected the CSF samples and clinical data. JC, YD, and TJ performed the experiments and analyzed the data. JC, YD, and HW wrote the manuscript and prepared the table/figures. All authors read and approved the final manuscript and agreed to submit it for publication.

### Conflict of interest statement

The authors declare that the research was conducted in the absence of any commercial or financial relationships that could be construed as a potential conflict of interest.

## Abbreviations

AD: Alzheimer's disease
CSF: Cerebrospinal fluid
CNS: Central nervous system
ELISA: Enzyme-linked immunosorbent assay
MS: Multiple sclerosis
mRS: modified Rankin Scale
NMDAR: N-methyl-D-aspartate receptor
NF-κB: Nuclear factor-kappa B
STAT3: Signal transducer and activator of transcription 3.
